# High Functioning Autism Spectrum Disorders in Adults: Consequences for Primary Caregivers Compared to Schizophrenia and Depression

**DOI:** 10.1007/s10803-017-3445-1

**Published:** 2018-01-08

**Authors:** Inge A. C. Grootscholten, Bob van Wijngaarden, Cornelis C. Kan

**Affiliations:** 1 Department of Psychiatry, Radboud University Medical Center, Nijmegen, Reinier Postlaan 4, 6525 EX Nijmegen, The Netherlands; 2 Netherlands Institute of Mental Health and Addiction, Da Costakade 45, 3521 VS Utrecht, The Netherlands; 3 Arnhem, The Netherlands

**Keywords:** Autism spectrum disorders, High functioning, Caregiver consequences, Parents, Spouses

## Abstract

Primary caregivers experience consequences from being in close contact to a person with autism spectrum disorder (ASD). This study used the Involvement Evaluation Questionnaire to explore the level of consequences of 104 caregivers involved with adults with High Functioning ASD (HF-ASD) and compared these with the consequences reported by caregivers of patients suffering from depression and schizophrenia. Caregivers involved with adults with an HF-ASD experience overall consequences comparable to those involved with patients with depression or schizophrenia. Worrying was the most reported consequence. More tension was experienced by the caregivers of ASD patients, especially by spouses. More care and attention for spouses of adults with an HF-ASD appears to be needed.

## Introduction

It has been recognized since the 1950s that a psychiatric disorder can have a major impact on the significant others (SO: parents, spouses, siblings, friends) of the patient (e.g. Schene 1990; Baronet 1999; Cuijpers and Stam 2000; Wittmund et al. 2002; Ostman et al. 2005; Gelkopf and Roe 2014). Most studies in the area of family caregiving have been conducted on SO concerned with patients with physical (e.g. Geurtsen et al. 2010) or mental disabilities (e.g. Murphy et al. 2007), dementia (e.g. Barusch and Spaid 1989) or psychiatric disorders such as schizophrenia or affective disorders (e.g. Goossens et al. 2008; van Wijngaarden et al. 2009; Granek et al. 2016; Kumar et al. 2015).

Autism spectrum disorders (ASD) are developmental disorders characterized by an impairment of reciprocal communication and social interactions and the presence of restricted stereotypical behaviors and interests (American Psychiatric Association 2013).

What is known about the impact of ASD on their SOs? Literature published on the impact of ASD on primary caregivers mainly focuses on the consequences for parents (mostly mothers) with an autistic child that often has a concomitant intellectual disability as well as a language disorder (e.g. Milgram and Atzil 1988; Hartley et al. 2011; Hayes and Watson 2013; Burke and Heller 2016; Tomeny 2016). These parents experience a profound number of consequences such as high levels of parenting stress (Lecavalier et al. 2006; Milgram and Atzil 1988) or feelings of guilt and failure (van Tongerloo et al. 2015). These levels of consequences are high compared to parents of children with a normal development, but also high compared to parents with a child with another disability such as Down syndrome, cerebral palsy, fragile X or Fetal Alcohol Spectrum Disorder (Hayes and Watson 2013). As a result of these chronic high levels of parenting stress these parents report an increase of mental health problems such as the risk of developing a depressive disorder (e.g. Tomeny 2016; Lin 2011; Lecavalier et al. 2006; Orsmond et al. 2007). See Tint and Weiss (2016) or Karst and Van Hecke (2012) for a recent review.

In the 1970s and 1980s it was thought that > 75% of ASD patients has an intellectual disability (Schalock et al. 2007). In more recent years, partly due to the changed perspective on autism as a spectrum disorder, more attention has been paid to those with ASD without an intellectual disability, called High Functioning ASD (HF-ASD). They have an average to above average intelligence (IQ > 70) and are often considered as having a ‘milder type’ of ASD (Baron-Cohen 2000).

Rao and Beidel (2009) have studied the impact on parents of children with HF-ASD, and concluded that in case of HF-ASD the levels of parenting stress, the restriction of family functioning, and the risk of psychological problems and poorer mental and physical health are higher compared to families with children without a psychiatric disorder. The higher intellectual functioning of the children did not ameliorate the high levels of stress in their parents. They also described that children with HF-ASD had less externalizing (behavioral) problems, but higher levels of internalizing problems, such as depression and anxiety, which would also contribute to the parental stress.

HF-ASD patients can often go unrecognized until well into adolescence or adulthood, possibly because they are able (at least to some extent) to compensate for social clumsiness by copying the behavior of their peers and because they are protected by structure and support from SO. But when changing circumstances (such as going to college, finding a job or becoming intimately involved) demand skills which exceed their abilities, they become aware of their inadequate coping and their deficits in social interaction (Marriage et al. 2009; Lehnhardt et al. 2013).

### What Do We Know About Adults with HF-ASD and the Impact on Their SO?

When focusing on adults with ASD, most literature is concerned with the problems of transition of the autistic child to adolescence and adulthood, difficulties with the different healthcare services organized for children and adults, and how this impacts the parents who continue to parent longer than parents with children without ASD (e.g. Cadman et al. 2012; Smith et al. 2012; Weiss et al. 2016; Hartley and Schultz 2015; Burke and Heller 2016), but these adults often had intellectual disabilities as well. Cadman et al. (2012) concluded that adolescence and young adulthood are associated with high levels of caregiver burden, that this burden was greater in ASD compared to attention-deficit/hyperactivity disorder and that this was mainly explained by the patient’s unmet needs.

Renty and Roeyers (2007) studied the marital satisfaction of 21 couples consisting of a HF-ASD male and a non-ASD female. They found that women whose husband showed less autism-specific traits reported higher levels of marital satisfaction. They also found that the marital adaptation in the men was significantly associated with more received and perceived social support from the spouse and from family and friends. Lau and Peterson (2011) studied the attachment styles of 22 adults with HF-ASD (7 males, 15 females) and found that 73% of them had an avoidant style of attachment, as opposed to the secured romantic attachment style found in 72% non-ASD spouses. Spouses of HF-ASD showed less marital satisfaction, but this could be explained by the presence of an ASD-child. They concluded that the husband’s or wife’s ASD status had little impact upon any aspect of marital quality.

In our clinical practice with adult patients newly diagnosed with HF-ASD we encounter primary caregivers who report a significant impact on their daily lives, relationships and family life. In order to assess the magnitude of this impact on the SO, we have acquired self-report data on the level of consequences and general aspects of their health in a sample of SO of adult HF-ASD patients, and have compared these data to data of samples of SO of patients with depression or schizophrenia. The reason we have compared our data to these two groups is because schizophrenia and major depressive disorders are both, although different in nature, considered as serious psychiatric disorders. HF-ASD is considered by some as a milder form of ASD, so it would be interesting to compare the consequences of HF-ASD for SO to these serious disorders.

For this study we hypothesized the following:

1. The impact on SO of adults with HF-ASD is comparable to the impact on SO of patients with depression or schizophrenia
2. More symptoms of ASD recognized by the SO correlate with a higher level of consequences reported by the SO
3. The impact on parents or spouses will be different
4. As in the case of schizophrenia and depression, SO who experience consequences of the disorder of their child/partner/family member will be at risk of developing (mental) health problems themselves

## Method

### Inclusion Criteria and Patient Samples

Data on the consequences of depression or schizophrenia on caregivers originate from the research done by van Wijngaarden et al. (2009). The depression sample consisted of relatives, friends, partners or other caregivers of patients who suffered from depression (major depressive disorder, dysthymic disorder, other depressive disorders). The patients were all treated in a mental hospital specialized in the treatment of depression in the Netherlands. Patients were asked for written permission to contact a SO and to send them a questionnaire by mail. From this data we used the outpatient data only (N = 237), to match our population.

The schizophrenia sample originated from the European EPSILON study (Becker et al. 2000), which identified a representative cohort of patients with schizophrenia. From this sample we used the outpatient data from the Northern European countries: the Netherlands, Denmark and the United Kingdom (N = 150). For more details see van Wijngaarden et al. (2009).

The ASD data were collected at the outpatient clinic of the Radboud University Medical Center in Nijmegen, the Netherlands. This clinic has a special outpatient program for adults suffering from developmental disorders (ASD and/or AD(H)D).

Adults referred to this department experience, for example, problems with social interaction, non-verbal communication, and/or restricted patterns of behavior and interests. These may cause problems in intimate and professional relationships, often resulting in social isolation, conflicts at work or within the family unit. The majority of these adults has not been diagnosed with an ASD during childhood due to their compensating normal to high intelligence (IQ > 70).

Patients who have been diagnosed with ASD were offered to participate in a psycho-educational group course. In this course information about ASD was given, and the impact of different aspects of ASD on an individual’s life was explored.

Every participant of the psycho-educational course had to bring a SO. This SO could be a spouse, a parent, a sibling, a friend, or a professional caregiver. At the Radboud Medical Center it is considered important that the information given in the psycho-educational course does not only reach the patient, but his/her surroundings as well. Someone who is close to the patient can be extremely helpful to the patient in recognizing the specifics of ASD in his/her own life, and can contribute to the generalization of the knowledge about ASD to everyday life beyond the group meetings.

Reasons for exclusion from the psycho-educational course were: intellectual disability (IQ < 70), concurrent psychosis, or a personality disorder of such an extent that it would interfere strongly with participation in a group with other patients. Patients who were excluded received individual psycho-education.

SO of ASD patients were asked to complete a number of questionnaires, including the Involvement Evaluation Questionnaire (IEQ), General Health Questionnaire (GHQ-12), and Autism-Spectrum Quotient (AQ), before the start of the psycho-educational course. Informed consent was signed by all the participants for use of data for research.

The Medical Ethics Committee of the Radboud University Medical Centre stated that no ethical approval was needed because of the non-invasive character of the self-report questionnaires.

### Measures

The *IEQ* is an internationally validated self-rating questionnaire which measures consequences experienced by those involved with people with psychiatric illnesses (van Wijngaarden et al. 2000, 2004, 2009; Martin et al. 2015; Cuijpers and Stam 2000). The IEQ-scores correlate to patients’ characteristics, and predict caregiver distress as assessed with the GHQ-12 (Goossens et al. 2008; Goncalves-Pereira et al. 2013). IEQ-scores assess various aspects of caregiving consequences such as the encouragement, supervision and care the caregiver has to provide to the patient, strain experienced on the relationship due to interpersonal problems between patient and caregiver, and to the caregiver’s worries. The core module measuring the ‘caregiving consequences’ comprises 31 items, which focus on the objective aspects of the caregiver’s experience, scored on 5-point Likert scales (0 = never, 1 = sometimes, 2 = regularly, 3 = often and 4 = always). The questionnaire is divided into four subscales: *Tension*, referring to a potentially strained interpersonal atmosphere between the patient and the SO; *Supervision*, which, amongst others, refers to the SO’s task of keeping the patient from committing dangerous acts, or the supervision of taking medication; *Worrying* refers to worrying about the patient’s health, safety and future, and *Urging* refers to the motivation and activation of the patient. The time-frame is the 4 weeks prior to the assessment. The IEQ has shown extensive reliability and internal consistency, in the Netherlands as well as internationally (Schene 1990; Becker et al. 1999, 2000; van Wijngaarden et al. 2000; Magne-Ingvar and Ojehagen 2005; Goossens et al. 2008; Geurtsen et al. 2010). It can be administered to any caregiver, including friends and neighbors, who are in contact with the patient for at least 1 h/week.

The * GHQ-12* is a well-validated self-administered instrument for the detection and measurement of psychopathology in the community (Goldberg et al. 1997; Goldberg and Williams 1988). It consists of 12 questions referring to subjects such as lack of sleep, loss of confidence, and feeling depressed. It is used to identify general emotional distress and possible health risks for the caregiver. Higher GHQ scores represent higher levels of distress. Possible answers are: ‘better than usual’, ‘as good as usual’, ‘worse than usual’, and ‘much worse than usual’ for positively phrased questions, and ‘not at all’, ‘same as usual’, ‘rather more than usual’, and ‘much more than usual’ for the negatively phrased questions.

The * AQ* was used to capture the severity of ASD symptoms. The AQ was developed by Baron-Cohen et al. (Baron-Cohen et al. 2001) and measures the amount of autistic traits in adults of normal intelligence. It has been broadly tested and widely used to differ between ASD and non-ASD caseness in the process of patient evaluation. The 50 item self-report questionnaire includes five domains: *Social skill, Attention switching, Attention to detail, Communication*, and *Imagination*. We used the ‘parent report version’; this can be completed by a parent or other primary caregivers (Baron-Cohen et al. 2006).

The Dutch AQ has shown good sensitivity and specificity (Hoekstra et al. 2008).

### Data Analysis

#### IEQ

In order to study the differences in IEQ subscale scores between the depression, schizophrenia and ASD samples, so-called ‘consequences indices’ (C-indices) were computed, as described by van Wijngaarden et al. (2009). IEQ item scores of ‘0’ or ‘1’ (never, sometimes) are considered as indicating ‘no real consequence’. Item scores of ‘2’ or higher (regularly, often or always) are considered as indicating a ‘real consequence’. The number of ‘real consequences’ are computed and divided by the total number of items in the scale. Four subscale C-indices and one overall C-index (total score), all ranging from ‘0’ (no consequences at all) to ‘1’ (maximum level of consequences) were computed this way.

#### GHQ-12

There are three different ways of scoring the GHQ-12: the binary (0,0,1,1), chronic (0,1,1,1) and Likert scoring (0,1,2,3) (Goldberg et al. 1997). The Likert scoring gives a wider and steadier score distribution to assess severity and can be used for correlation analysis. The Likert scoring was therefore used in this study. The possible score ranges from 0 to 36, with higher scores representing higher levels of distress. Threshold for caseness varies in different populations studied (Goldberg et al. 1998). As no threshold was published for the Dutch population, the threshold for the German population as published by Schmitz et al. (1999) was used (11/12). We expected Germany, being a neighboring country of the Netherlands with approximately the same socio-economic situation and organization of health care, to be the best comparable country available.

#### AQ

There are two ways of scoring the AQ: a 4-point Likert score or a binary one in which answers ‘definitely agree’ and ‘agree’ score as 1 on autism positive traits and ‘definitely disagree’ and ‘disagree’ score as 1 on autism negative traits. The binary score is proposed by Baron-Cohen and is used in this research. It has a minimum of 0 and a maximum score of 50, the higher indicating more autistic traits. The cut-off for caseness was 32 as proposed by Baron-Cohen et al. (2001).

### Statistics

The statistical calculations were made using the IBM Statistical Package for the Social Sciences version 22.0. Comparisons between IEQ C-indices scores for the different disorders and SO-groups were made by the Kruskal–Wallis test for non-parametrically distributed, independent samples as advised by Field (2013). Comparison between GHQ scores were made by the Mann–Whitney test. All effects are reported at a significance level of p < .05, unless stated differently. Effect sizes were computed using Pearsons’ r. Correlation of IEQ with GHQ scores and of IEQ with AQ scores were calculated using the Spearman rank correlation coefficient (two-tailed) for non-parametrically distributed variables. Two-tailed ANOVA was used to compare AQ data in the SO-groups. We did not control for any demographic factors throughout the analysis.

## Results

Between 2006 and 2009 approximately 660 patients were referred to the outpatient clinic for diagnostic assessment of ASD. 38.5% of these 660 patients was not diagnosed with an ASD, 17% was classified with autistic disorder, 19% with Asperger’s disorder, and 21% with PDD-NOS by use of Diagnostic and Statistical Manual of Mental Disorders (DSM-IV TR) (American Psychiatric Association 1994). 1.5% of the referred patients did not finish the course of examination. No files were found in the archives with respect to 3% of the patients.

129 of the 376 patients diagnosed with ASD enrolled in the psycho-educational group course. There were several reasons, apart from not meeting the inclusion criteria, for patients not to participate in the psycho-educational group, ranging from unwillingness to participate in a group or living too far away from the center, to having the opinion that a psycho-educational group could do nothing to alleviate his/her problems. Bi-nominal regression analysis showed that there was no selection bias, based on sub-classification according to DSM-IV, and age or gender of the patient.

Of the 129 SO who joined a patient in the psycho-educational group course between 2006 and 2009, 110 handed in the questionnaires. Due to missing data, questionnaires of 104 SO could be used for analysis.

### Subject Characteristics

Table 1 shows socio-demographic characteristics of patients and SO for ASD, compared to schizophrenia and depression. SO who were not a parent or a spouse (e.g. a sibling, friend, child, neighbor, colleague, or coach) are combined in the group named ‘other’ because of the low number of SO in each of these remaining groups, and the comparable level of consequences experienced by these SO.

**Table 1:** Socio-demographic characteristics of patients and caregivers by diagnostic group

	Depression N = 237		Schizophrenia N = 150		ASD N = 104	
Gender of patient	62.0% female		34.7% female		20.2% female	
Gender of SO	51.9% female		70.7% female		81.7% female	
Age of patient Mean (SD)	44.4 (13.0)		37.8 (11.8)		39.9 (14.0)	
Age of SO Mean (SD)	45.7 (13.1)		53.2 (13.9)		48.2 (11.3)	
Hours of contact per week > 32	70.9%		33.3%		47.1%	
Live in same household	79.3%		37.3%		72.1%	

Relationship between patient and SO	N	%	N	%	N	%
SO is parent	17	7.2	87	58.0	31	29.8
Mother	11		65		25	
Father	6		22		6	
SO is spouse	173	73.0	16	10.7	59	56.7
Wife	77		8		50	
Husband	96		8		9	
SO is other	47	19.8	47	31.3	14	13.5
Sibling	16		21		5	
Child	12		6		1	
Other family member	3		4		–	
Friend/colleague	15		11		5	
Professional caregiver	1		5		3	

Part of this data was used in van Wijngaarden et al. (2009)

*ASD* Autism spectrum disorder, *SO* significant other

There were several substantial differences between the three groups concerning the age and gender of patients and SO. The depression sample consisted mostly of spouses who lived in the same household and were of approximately the same age as the patients. Of the three samples they spent the most time together. The schizophrenia sample consisted mostly of mothers who were the main caregivers of their schizophrenic sons. The ASD sample consisted mostly of spouses, but there was a gender difference in comparison to the depression sample: most of the patients in the ASD sample were males, and most of the SO were females, while in the depression sample the gender distribution was almost 50–50. These differences in gender distribution can be explained by the specific gender distribution of the disorder: ASD is diagnosed at least three times as often in males as in females (Muhle et al. 2004; Loomes et al. 2017).

### IEQ Scores

Our sample was too small to perform a reliable confirmatory factor analysis. In order to get some idea of the validity of the IEQ for an ASD sample, a preliminary exploratory factor analysis was conducted. It showed a slightly different factor structure compared to the standard IEQ structure. We found four subscales, which consisted of *Tension, Worrying, Urging* and *Supervision* combined together, and a fourth mixed scale. *Tension* showed to be the most robust factor. Five items referring to *Supervision* and *Urging* were not included in one of the factors.

In order to test the reliability of the IEQ in the ASD-population, Crohnbach’s alpha was computed, using the standard IEQ scales. These were acceptable for the total score (0.8), the subscale *Tension* (0.8), subscale *Worrying* (0.7) and subscale *Urging* (0.7). Subscale *Supervision*, however, had an alpha of 0.3. Because this subscale is not reliable in our sample it was not used in the comparison analysis.

Caregiver consequences in the depression, schizophrenia and ASD sample were compared using the total score and the subscales *Tension, Worrying*, and *Urging*. In Table 2 the mean C-indices of the IEQ scales for the three samples are presented. No difference was found for the total consequences. On the subscale of *Tension*, however, there was a significant difference between the three samples [H(2) = 19.76 p < .000]. Pair-wise comparison showed that *Tension* was reported significantly more often by the SO of ASD, compared to the schizophrenia (effect size r = 0.28) and depression sample (r = 0.17).

**Table 2:** Mean C-index IEQ-scores for caregivers by diagnostic group

IEQ subscales	Depression	Schizophrenia	ASD	p*
	N = 237	N = 150	N = 104	
	Mean C-index (SD)	Mean C-index (SD)	Mean C-index (SD)	
Tension	0.16 (0.21)	0.12 (0.17)	0.24 (0.23)	< .000
Worrying	0.31 (0.31)	0.36 (0.31)	0.34 (0.26)	.162 (n.s.)#
Urging	0.15 (0.19)	0.21 (0.26)	0.17 (0.19)	.079 (n.s.)
Total score	0.17 (0.16)	0.18 (0.17)	0.19 (0.14)	.076 (n.s.)

Part of this data was used in van Wijngaarden et al. (2009)

*ASD* Autism Spectrum Disorder, *IEQ* Involvement Evaluation Questionnaire

*Kruskal Wallis for independent samples

^#^n.s.: non-significant p > .0 5

### GHQ-12 Scores

A score above threshold (11) indicates psychiatric morbidity or severe emotional distress. The mean score on the GHQ-12 was 12.56 for the SO in the schizophrenia sample, and 14.56 for the SO in the ASD sample, which where both clearly above the threshold (Table 3). In fact, 49% of the SO of schizophrenia and 62% of SO of ASD patients scored above the threshold of 11 and were therefore at risk of developing health problems themselves. The differences between the SO in both samples were significant. SO of ASD patients experienced significantly more emotional distress compared to SO of schizophrenic patients [U = 9130.00, p = .006, r = 0.18]. Also, there was a significant positive correlation between the IEQ (subscales and overall-score) and the GHQ-12 scores, meaning that higher levels of consequences are correlated with more distress. No GHQ-12 data were available for the depression sample.

**Table 3:** Correlation between GHQ-12 and IEQ for caregivers of schizophrenia and ASD

	GHQ-12 schizophrenia	GHQ-12 ASD	p*
Mean score (SD)	12.56 (6.36)	14.56 (6.23)	.006
GHQ-12 score > 11	48.6%	61.8%	

IEQ subscales	Correlation coefficient	Correlation coefficient	
Tension	.430**	.533**	
Worrying	.347**	.402**	
Urging	.272**	.317**	
Total score	.407**	.531**	

*ASD* Autism Spectrum Disorder, *GHQ* General Health Questionnaire, *IEQ* Involvement Evaluation Questionnaire

*Mann Whitney U

**p (2-tailed) < .001 p value and correlation coefficient were calculated using Spearman’s rho

### Looking Closer at Caregivers Confronted with ASD:

#### IEQ

In Table 4 the mean C-indices of the IEQ scales for the different groups of SO connected to a patient with ASD are presented. Significant differences between the groups of primary caregivers were found on all three subscales and on the total consequences score. Differences were greatest on subscale *Tension* [H(2) = 20.98 p < .000]. Pairwise comparison showed that spouses scored significantly higher than both parents (p adj = .008 r = 0.31) and others (p adj < .000 r = 0.48).

**Table 4:** Mean C-index IEQ-scores and GHQ-12 scores for parents, spouses and other caregivers in ASD

IEQ subscales	ASD	ASD	ASD	p*
	Parent	Spouse	Other	
	Mean (SD)	Mean (SD)	Mean (SD)	
	N = 31	N = 59	N = 14	
Tension	0.16 (0.17)	0.32 (0.24)	0.06 (0.15)	< .000
Worrying	0.44 (0.25)	0.32 (0.24)	0.21 (0.30)	.011
Urging	0.22 (0.18)	0.16 (0.19)	0.09 (0.16)	.024
Total score	0.20 (0.13)	0.21 (0.14)	0.10 (0.13)	.018
GHQ-12	13.87 (4.87)	15.91 (6.68)	10.15 (4.93)	.002

*ASD* Autism Spectrum Disorder, *GHQ* General Health Questionnaire, *IEQ* Involvement Evaluation Questionnaire

*p Value was calculated using Kruskal–Wallis for independent samples

Parents scored higher on subscale *Worrying* compared to other caregivers (p adj = .012 r = 0.43), but the difference between parents and spouses was non-significant.

Parents scored higher on subscale *Urging* compared to other caregivers (p adj = .025 r = 0.39), but in this case also, differences between parents and spouses were non-significant.

Both parents and spouses scored higher on total consequences score than other caregivers (parents vs. others p adj = .039, r = 0.37, spouses vs. others p adj = .017 r = 0.32). Also in this case, parents and spouses did not differ.

#### GHQ-12

Table 4 shows that parents and spouses have higher GHQ-12 scores compared to other caregivers (parents vs. others p adj = .05, r = 0.36, spouses vs. others p adj = .001 r = 0.40), but the difference between parents and spouses was non-significant.

#### AQ Scores

The mean score on the AQ was 34 (SD = 7), well above the cut-off for caseness of 32 points (Table 5).

**Table 5:** Mean AQ-scores scores for parents, spouses and other caregivers in ASD

	Total	Parent	Spouse	Other	p*
	N = 104	N = 31	N = 59	N = 14	
Total score AQ	34.03 (7.03)	33.25 (6.72)	34.24 (7.67)	34.95 (4.11)	.904 (n.s.)^#^
Social skill	7.80 (1.98)	7.61 (2.35)	7.89 (1.83)	7.82 (1.74)	.907 (n.s.)
Attention switching	7.87 (1.83)	7.70 (1.78)	7.80 (1.95)	8.58 (1.16)	.707 (n.s)
Attention to detail	5.24 (2.45)	5.36 (2.18)	4.95 (2.62)	6.42 (1.98)	.612 (n.s.)
Communication	6.63 (2.09)	6.54 (2.11)	6.75 (2.08)	6.25 (2.22)	.846 (n.s.)
Imagination	6.50 (2.15)	6.04 (2.26)	6.85 (2.08)	5.88 (2.02)	.253 (n.s.)

*ASD* Autism Spectrum Disorder, *AQ* Autism-spectrum Quotient

*Kruskal–Wallis test for non-parametric data

^#^n.s.: non significant p > .05

Cronbach’s alpha for the subscales was medium to acceptable: *Social skill*: 0.7, *Attention switching*: 0.6, *Attention to detail*: 0.7, *Communication*: 0.6, *Imagination*: 0.6. The Kruskal–Wallis test showed no significant difference in the AQ score between the different SO groups. There was no significant correlation between the total amount of autistic traits recognized by the SO and the consequences assessed with the IEQ (Table 6).

**Table 6:** Correlation between total AQ score and mean C-index IEQ-scores

		C-index tension	C-index worrying	C-index urging	C-index total score
Total score AQ	Spearman’s rho	.042	.140	− .070	.048
	p (2-tailed)	.716	.221	.543	.676

*AQ* Autism-spectrum Quotient, *IEQ* Involvement Evaluation Questionnaire

## Discussion

The aim of this study was to investigate the consequences experienced by SO of adult HF-ASD patients and to compare these with the consequences experienced by SO of patients with depression or schizophrenia. The main outcome of this study is that the overall consequences experienced by the SO of adult HF-ASD patients are comparable to those of patients with depression or schizophrenia. On *Tension* they even score significantly higher than SO in the other two samples. This is an important result, because HF-ASD in adults is often not recognized or diagnosed, and support and treatment for ASD patients and their SO have never been a major focus of attention in mental health. Also, HF-ASD is sometimes regarded as a milder form of ASD (Baron-Cohen 2000). Nonetheless, the consequences, especially for relationships, can be substantial. This is consistent with our findings in the GHQ-12 data, which show that primary caregivers of HF-ASD patients experience profound emotional distress, and are beyond average at risk of developing mental health problems themselves. These patients and their SO have been feeling isolated, neglected and poorly understood, and have therefore not been receiving the support they needed.

We found that parents and spouses experience significantly more consequences than other ASD caregivers. This can be explained by the more intense and emotional bonding between a spouse or parent and the patient, the large amount of time they spend together in a household, and the different expectations that the caregiver has of their spouse or child, compared to a more distant relationship. Other researchers also found that the amount of time spent together/living together with the patient is an important predicting factor on experienced consequences (van Wijngaarden et al. 2009; Ostman et al. 2005; Martin et al. 2015; Kronenberg et al. 2016).

### Specifics of ASD as Hypothetic Explanations for the Consequences Found

Because of the high levels of heritability of autistic traits (Muhle et al. 2004; Hoekstra et al. 2007), SO of ASD patients have a higher risk of having another family member affected by ASD than the general population (van Steijn et al. 2012). This is recognized as an additional cause of stress in mothers with multiple children with ASD (Orsmond et al. 2007; Ekas et al. 2009). This may also be the case for spouses who, besides having a partner with ASD, have one or more children with ASD or another disability (van Steijn et al. 2014; Lau and Peterson 2011).

Parents of adults with HF-ASD reported high levels of worrying. It is known from research concerning parents of young children that parenting a child with ASD can cause high levels of parenting stress and is correlated with depression in mothers, but it was believed that this stress diminished as the child grew older (Lounds et al. 2007; Ekas and Whitman 2010; Hartley et al. 2011). Our findings show that parents continue to worry profoundly about their children with ASD, even when they have reached adulthood. This is consistent with the research by Cadman et al. (2012) who found that caregivers of adolescents and young adults with ASD had very high levels of burden, a level that was comparable to caregivers of persons with an acquired brain injury.

Spouses of HF-ASD patients experience more tension in their relationship with the patient compared to parents. When a partner becomes a patient, more role-taking confusion occurs than when the patient is a child. Spouses sometimes talk about their mentally ill partner in terms of “having another child” (Wittmund et al. 2002). This is confirmed by Ostman et al. (2005) and Cuijpers and Stam (2000) who also found that spouses showed increased burden compared to parents and others.

Based on our clinical experience, the lack of mutual reciprocal interaction, one of the main disabilities in patients with HF-ASD, is probably a major additional factor contributing to the perceived burden of the spouses. Due to the ability to ‘feel’ and understand the other person’s feelings and needs, and responding accordingly, reciprocal interaction is valued by many as essential for a fulfilling intimate relationship. This requires a certain amount of ‘theory of mind’, often lacking in patients with ASD (Baron-Cohen 1991).

We believe parents are less burdened by this, because of the difference in expectations that a parent has of a child compared to the expectations one has of a spouse or partner (van Tongerloo et al. 2015).

Another explanation for the higher levels of tension reported by spouses of HF-ASD patients is that of gender: Wittmund et al. (2002) found that female spouses of psychiatric patients were at a higher risk of getting depressed themselves than male spouses. In the ASD-sample, 85% of the spouses was female (50 women vs. 9 men), which reflects the gender distribution of ASD (Loomes et al. 2017). Other research is inconclusive in regard to the question whether gender is associated with the amount of caregiver distress (Sharma et al. 2016; Baronet 1999).

Assortive mating (non-random partner choice) might influence the relationship between an HF-ASD patient and spouse. Baron-Cohen (2006) suggested that assortive mating between two high systemizers (people with a high preference to lawfulness, predictability and categorizing information) would lead to more autistic children, but Hoekstra et al. (2007) found no evidence for assortive mating with respect to mutual autistic traits. It is unclear to what extent assortive mating might influence the consequences experienced by a spouse of an HF-ASD patient. One could suggest that spouses who have several autistic traits themselves (broad phenotype) would be less bothered or burdened by the social handicaps of their autistic partners. On the other hand, when someone is feeling overwhelmed by an outside world that they do not completely understand themselves, the strain of caring for someone with the same problems could be experienced as burdensome.

Contrary to the assortive mating theory, HF-ASD subjects might also be attractive to empathic, supporting partners who feel an urge to care for them, and compensate for their limitations, or to partners who have been emotionally neglected in their youths and tend to choose a partner who repeats this emotional neglecting. However, receiving no emotional reciprocity in return might drain and strain them in the long run.

Literature is inconsistent on whether ASD symptom severity contributes to levels of consequences (Ricard et al. 1999; Tint and Weiss 2016). Mothers often report higher levels of consequences when children with ASD show more disruptive behavior or higher ASD symptom severity (Ekas and Whitman 2010; Tomeny 2016). Also, Renty and Roeyers (2007) found that spouses of men with ASD reported higher levels of marital satisfaction when their husbands showed less autism-specific traits as measured with the AQ. In our sample, the level of ASD symptoms recognized by the SO was not correlated to the level of consequences they experienced. Reliability of the AQ domains in our sample was comparable to that of Hoekstra et al. (2008).

To our knowledge this is the first time that the caregiver consequences for those involved with an adult with HF-ASD have been examined using the IEQ. Even though most patients in our clinic had not been diagnosed with ASD during childhood, the impact of their limitations on their surroundings was substantial.

Because of the consequences experienced by spouses of patients with HF-ASD, as found in this study and voiced in the psycho-education group meetings, we believe that extra attention is needed for this group. They experience a lot of interpersonal strain in their relationships and are at risk of developing mental health problems themselves.

### Limitations of This Study

The IEQ has been validated in schizophrenia and depression samples, and has been used with many different psychiatric disorders since, but it has not officially been validated yet with respect to ASD. Cronbach’s alpha was acceptable for the overall score and for 3 of the 4 sub-scales but not for the subscale *Supervision*. Items in this subscale, like “how often during the past 4 weeks have you guarded your relative/friend from committing dangerous acts” or “have you guarded your relative/friend from taking illegal drugs” are possibly not as applicable to HF-ASD as they are to depression or schizophrenia. These were also items that could not be included in one of the factors of the preliminary exploratory factor analysis, which showed a slightly different factor structure compared to the standard IEQ structure. *Tension* seems to be an issue in ASD, which confirms the results described in this article.

Even though a Confirmatory Factor Analysis was not performed due to the small sample size, it is encouraging to see that the IEQ in ASD seems to have a reasonable amount of reliability and validity. A larger sample size is needed for official validation for the IEQ in ASD. Also, more studies need to be carried out to replicate our findings.

Another limitation concerns the different backgrounds and characteristics of the samples, which reflect the different context in which the caregiving takes place, and also reflects the age and gender difference between the disorders. The ASD data were collected from SO who joined a psycho-educational ASD course in a specialized outpatient clinic. Not all patients diagnosed with ASD participated in this group, and because of the different reasons for not participating we cannot say for sure in which direction a selection bias has influenced our results. However, all data were from SO who were in close contact with a patient, and experienced consequences more or less on a daily basis.

ASD patients were diagnosed between 2006 and 2009, the DSM IV-TR (American Psychiatric Association 1994) version was used for classification. As the DSM-IV sub-classifications are no longer applicable in DSM 5 (American Psychiatric Association 2013), we have only reported the frequencies of these sub-classifications and have not included them in further analyses.

Recent studies (Buck et al. 2014; Cawthorpe 2017; De-la-Iglesia and Olivar 2015; Hofvander et al. 2009; Orinstein et al. 2015; Cadman et al. 2012) suggest that co-morbid psychiatric conditions (e.g. depression) make life more difficult especially for adults with HF-ASD. This would probably also be of impact on the SO and of influence on the consequences they experience. Unfortunately, no data concerning co-morbid psychiatric disorders are available for the HF-ASD sample, nor for the depression and schizophrenia sample.
